# Conventional reversal of rocuronium-induced neuromuscular blockade by sugammadex in Korean children: pharmacokinetics, efficacy, and safety analyses

**DOI:** 10.3389/fphar.2023.1127932

**Published:** 2023-04-13

**Authors:** Sang-Hwan Ji, Ki Young Huh, Jaeseong Oh, Hee-Jeong Jeong, Young-Eun Jang, Eun-Hee Kim, Ji-Hyun Lee, Jin-Tae Kim, Hee-Soo Kim

**Affiliations:** ^1^ Seoul National University College of Medicine, Seoul, Republic of Korea; ^2^ Department of Anesthesiology and Pain Medicine, Seoul National University Hospital, Seoul, Republic of Korea; ^3^ Department of Clinical Pharmacology and Therapeutics, Seoul National University Hospital, Seoul, Republic of Korea

**Keywords:** anesthesia, pediatric, pharmacodynamics, pharmacokinetics, sugammadex, rocuronium

## Abstract

**Background:** Sugammadex is known to reverse neuromuscular blockade induced by non-depolarizing agents. In children, the recommended dose for reversal of moderate neuromuscular blockade is 2 mg/kg. We investigated the pharmacokinetics and pharmacodynamics of sugammadex in Korean children.

**Methods:** Children (2–17 years of age) undergoing brain or spine surgery were enrolled and randomly assigned to control (neostigmine) and 2, 4, or 8 mg/kg sugammadex groups. Following induction of anesthesia and monitoring of the response to train-of-four stimulation, 1 mg/kg rocuronium was intravenously administered. Upon reappearance of the second twitch to train-of-four stimulation, the study drug was administered according to group allocation. The plasma concentrations of rocuronium and sugammadex were serially measured at nine predefined time points following study drug administration. To determine efficacy, we measured the time elapsed from drug administration to recovery of T_4_/T_1_ ≥ 0.9. For pharmacokinetics, non-compartmental analysis was performed and we monitored adverse event occurrence from the time of study drug administration until 24 h post-surgery.

**Results:** Among the 29 enrolled participants, the sugammadex (2 mg/kg) and control groups showed recovery times [median (interquartile range)] of 1.3 (1.0–1.9) and 7.7 (5.3–21.0) min, respectively (*p* = 0.002). There were no significant differences in recovery time among the participants in sugammadex groups. The pharmacokinetics of sugammadex were comparable to those of literature findings. Although two hypotensive events related to sugammadex were observed, no intervention was necessary.

**Conclusion:** The findings of this pharmacokinetic analysis and efficacy study of sugammadex in Korean children indicated that sugammadex (2 mg/kg) may be safely administered for reversing moderate neuromuscular blockade. Some differences in pharmacokinetics of sugammadex were observed according to age.

**Clinical Trial Registration:** http://clinicaltrials.gov (NCT04347486)

## 1 Introduction

In the field of anesthesia, it is well established that sugammadex can effectively reverse neuromuscular blockade induced by steroidal non-depolarizing neuromuscular blocking agents. Sugammadex binds to rocuronium by forming a one-to-one complex (Gijsenbergh et al., 2005), without causing muscarinic adverse effects or residual blockade, which can be a potential risk for patients when administering neostigmine (Gijsenbergh et al., 2005; Sorgenfrei et al., 2006).

During administration of general anesthesia for surgery, the degree of neuromuscular blockade is peripherally monitored, generally via train-of-four stimulation, in which 50 mA of electrical stimulation is applied to the peripheral nerves (Ali et al., 1970), primarily the ulnar nerve. Neuromuscular blockade is typically considered a “moderate” blockage, which is suitable for most types of surgeries, when the train-of-four count is 2. To reverse this degree of blockage, 2 mg/kg sugammadex is recommended for both adults and children (Sorgenfrei et al., 2006).

The pharmacokinetics of sugammadex in adult patients have been well reported, in which it is characterized by a linear relationship up to 16 mg/kg and a volume of distribution of 11–14 L (Keating, 2016). Sugammadex is mostly eliminated via renal excretion with an elimination half-life of approximately 2 h (Keating, 2016). Accordingly, the clearance of sugammadex is significantly reduced in patients with renal failure (Staals et al., 2010). In addition, ethnicity is associated with the pharmacokinetics of sugammadex (Keating, 2016).

However, there have been comparatively few pharmacokinetic studies of sugammadex in the pediatric population. Among those that have been conducted, the findings of an early study involving eight infants, 22 children, 28 adolescents, and 26 adults indicated that the efficacy of sugammadex was comparable among the different age groups (Plaud et al., 2009). However, due to the sparsity of suitable samples, the associated pharmacokinetics could not be sufficiently assessed (Plaud et al., 2009). Although several randomized clinical trials suggested comparable efficacy and safety profiles in pediatric and adult patients (Plaud et al., 2009; Ozgun et al., 2014; Won et al., 2016; Ammar et al., 2017; Liu et al., 2017; Matsui et al., 2019; Voss et al., 2022), precise evaluation would be necessary for specific dose adjustment in the pediatric population.

Therefore, we conducted a randomized clinical trial to evaluate the pharmacokinetics, efficacy, and safety of sugammadex in the context of reversing moderate neuromuscular blockade in Korean children. The study was designed to complement a previous study by our study group, in which we evaluate the pharmacokinetics, efficacy, and safety of sugammadex in the context of intense neuromuscular blockade (Ji et al., 2023). In the present study, we sought to evaluate whether a 2 mg/kg dose of sugammadex could effectively reverse neuromuscular blockade on reappearance of T_2_ in response to train-of-four stimulation.

## 2 Methods

### 2.1 Study design and population

This study was designed as a randomized, controlled, single-blinded exploratory study comparing groups administered different doses of sugammadex, and was conducted in a single tertiary hospital located in Seoul, Republic of Korea. The study adhered to the tenets of the Declaration of Helsinki (revised in 2013), and the protocol was approved by the Institutional Review Board of Seoul National University Hospital (no.: 2002-148-1105, approval date: 03/25/2020) and the Ministry of Food and Drug Safety of the Republic of Korea (no.: 32,828, approval date: 04/13/2020). The study was registered at http://clinicaltrials.gov (NCT04347486, principal investigator: Hee-Soo Kim, published date: 04/14/2020) and was conducted in accordance with the Good Clinical Practice guidelines of the International Council for Harmonization and the Declaration of Helsinki. Participants were recruited between April 2020 and December 2021.

Children aged between 2 and 17 years who were scheduled to undergo elective brain or spinal surgery under general anesthesia were enrolled. Written informed consent was obtained from the parent(s) of each participant, as well as from the participants older than 6 years of age. The exclusion criteria were a history of hypersensitivity to any anesthetic agent, presence of cardiovascular or urological disease, presence of renal or hepatic impairment, use of a neuromuscular blocking agent or drugs that can affect the action of neuromuscular blocking agents prior to anesthetic induction, a history of malignant hyperthermia, anticipation of massive hemorrhage during surgery, and the refusal of one or both parents or legal guardians to permit enrollment.

### 2.2 Study protocol

When the participants arrived at the operating room, non-invasive blood pressure, oxygen levels (determined using pulse oximetry), and heart activity (determined using electrocardiography) were monitored. Anesthesia was induced by intravenous (IV) injection of sodium thiopental or propofol. After loss of consciousness, anesthesia was maintained with the continuous IV infusion of an opioid and propofol. To monitor neuromuscular blockade, a ToFscan^®^ device (IDMED, Marseille, France) was attached to each participant’s unilateral ulnar nerve. Train-of-four stimulation, which applies four twitch stimulations with an intensity of 50 mA and frequency of 2 Hz, was commenced and repeated at 15-s intervals until the end of surgery. The responses were measured via acceleromyography and automatically recorded on a computer using a vital recorder (Lee and Jung, 2018). Thereafter, 1 mg/kg of rocuronium was administered IV, followed by arterial catheterization at an extremity for continuous monitoring of invasive blood pressure. On the reappearance of the second twitch (T_2_) in response to the train-of-four stimulation, the study drug was administered IV.

### 2.3 Randomization and blinding

Allocation of patients to treatment groups was determined based on a randomization table obtained from https://sealedenvelope.com/. Having enrolled participants, they were allocated to one of the following groups: a control group (0.03 mg/kg neostigmine) or groups treated with 2, 4, or 8 mg/kg sugammadex. The study groups were assembled by a single anesthesiologist. The participants and their parents were blinded to group allocation.

### 2.4 Pharmacokinetic measurements

Nine arterial blood samples were collected for pharmacokinetic measurements: 2 min after rocuronium injection, and immediately prior to and at 2, 5, 15, 60, 120, 240, and 480 min post-administration of the study drug. For each sampling time point, the plasma concentrations of rocuronium and sugammadex were measured. In all groups, the concentration of sugammadex was not measured at the first time point and at all points in the control group. When sampling was not performed at the exact scheduled time, the actual sampling time was recorded (Choi et al., 2013).

### 2.5 Measurement of plasma concentrations

At each pharmacokinetic sampling point, 1 mL of arterial blood was withdrawn and immediately stored in a sodium heparin tube (BD Vacutainer^®^ sodium heparin [N] 75 USP Units; Becton Dickinson Korea, Seoul, Korea). After centrifuging at 3,000 rpm (1,167 × *g*) for 10 min, the supernatant was stored in a sterile internal cryogenic vial (Cryotain™; SCILAB Korea, Seoul, Korea). The collected specimens were stored in a freezer below −70°C until analysis. The plasma concentrations of sugammadex and rocuronium were determined using liquid chromatography–tandem mass spectrometry. The assays were conducted in compliance with Good Laboratory Practice regulations. As this assay cannot be used to discriminate the sugammadex–rocuronium complex from the free forms of these drugs, all plasma concentrations were considered total plasma concentrations. The internal standards used for sugammadex and rocuronium were phenformin-d_5_ and rivastigmine-d_4_ (Toronto Research Chemicals, North York, Canada), respectively.

The assay methods for rocuronium and sugammadex were validated previously and partial validation was performed based on a risk-based approach (Farenc et al., 2001; de Zwart et al., 2011; Briggs et al., 2014; de Moraes et al., 2014). Calibration curves for rocuronium were established in the ranges of 100–10000 ng/mL (*r* = 0.9965) and 2–200 ng/mL (*r* = 0.9980). Accuracy was between −3.0% and −1.0%, −8.8 and −7.5 in the range of 100–10000 and 2–200 ng/mL, respectively. The corresponding precisions were 2.0%–10.0% and 2.8%–5.0%, respectively. Accuracy and precision of the diluted samples were −1.0% and 7.0%. Carryover effect was not detected.

The assay method for sugammadex was also validated. Calibration curve for sugammadex was established in the range of 0.1–100 μg/mL (*r* = 0.9981). Accuracy was between −2.7% and −1.0% and precision was between 1.7% and 6.2%. Accuracy and precision of the diluted samples were 6.0% and 4.7%, respectively. Carryover effect was not detected.

### 2.6 Pharmacokinetic assessments

The pharmacokinetic parameters of sugammadex and rocuronium were calculated based on non-compartmental analysis via Phoenix WinNonlin (Version 8.1; Certara United States, Princeton, NJ, United States). The maximum plasma concentration (C_max_) and the time to reach C_max_ (T_max_) were obtained directly based on the observed values. The area under the plasma concentration-time curve (AUC) to the last measurable concentration (AUC_last_) was calculated using the linear up-log down trapezoidal method. AUC extrapolated to infinity (AUC_inf_) was calculated as the sum of AUC_last_ and the last measurable concentration divided by the elimination phase constant (λ_z_), which was regressed on the log-transformed concentration. The terminal phase half-life (t_1/2_) was calculated as the natural logarithm of 2 divided by λ_z_. The volume of distribution and clearance were calculated as dose/(λ_z_ × AUC_inf_) and dose/AUC_inf_, respectively. Dose-normalized C_max_, AUC_last_, and AUC_inf_ values were calculated as each parameter divided by the administered dose. We compared the obtained pharmacokinetic parameters with those of our previous study for reversal of intense neuromuscular blockade (Ji et al., 2023).

### 2.7 Pharmacodynamic measurements

Responses to train-of-four stimulation were continuously monitored at 15-s intervals. When the number of twitches was fewer than four, only the number of twitches was recorded. After the appearance of the fourth twitch, the ratio of the fourth twitch to the first twitch (T_4_/T_1_) was recorded, as this is considered to represent receptor occupancy by rocuronium. As a T_4_/T_1_ ratio ≥90% is considered sufficient for extubation, the time from administration of the study drug to recovery of a T_4_/T_1_ ratio ≥90% was taken to be the primary pharmacodynamic outcome.

### 2.8 Monitoring of safety

Electrocardiogram, mean blood pressure, pulse oximetry, and body temperature were monitored until 24 h after the end of surgery. We monitored the occurrence of any changes >30% of the baseline in heart rate or mean blood pressure, pulse oximetry of <92%, changes in body temperature to above 38.3°C or below 35.5°C, and other complications, including nausea, vomiting, urticaria, and any anaphylactic reactions.

### 2.9 Statistical analysis

Given the exploratory nature of the study, sample size was calculated empirically. We allotted eight children to each group on the basis of previously conducted pediatric pharmacokinetic studies for sugammadex that had assessed group sizes of 4–10 children (Plaud et al., 2009; Voss et al., 2022). Normality was assessed using the Shapiro–Wilk test. Baseline characteristics and recovery times of patients in the different study groups were compared using analysis of variance for normally distributed data, and the Kruskal–Wallis test with *post hoc* analysis using the Mann–Whitney *U* test with Bonferroni correction for non-normally distributed data. Analyses were performed using R ver. 4.0.3 (The R Foundation, http://r-project.org), with the acceptable alpha error rate set at 0.05.

For non-compartmental analysis, subgroup analysis was done for C_max_, AUC_last_, and AUC_inf_.: parameters of rocuronium were compared between each dose group and parameters of sugammadex were compared between age group classified according to a recent pharmacodynamics study (Voss et al., 2022) using the Kruskal–Wallis test. Non-parametric *post hoc* evaluation was performed with the Dwass, Steel, and Critchlow–Fligner procedures. Parameters from non-compartmental analyses of this study and our previous study (Ji et al., 2023) were compared for both drugs using Student’s *t*-test or Wilcoxon rank sum test. These analyses were done via SAS software (version 9.3; SAS Institute, Inc., Cary, NC, United States).

## 3 Results

**FIGURE 1:**
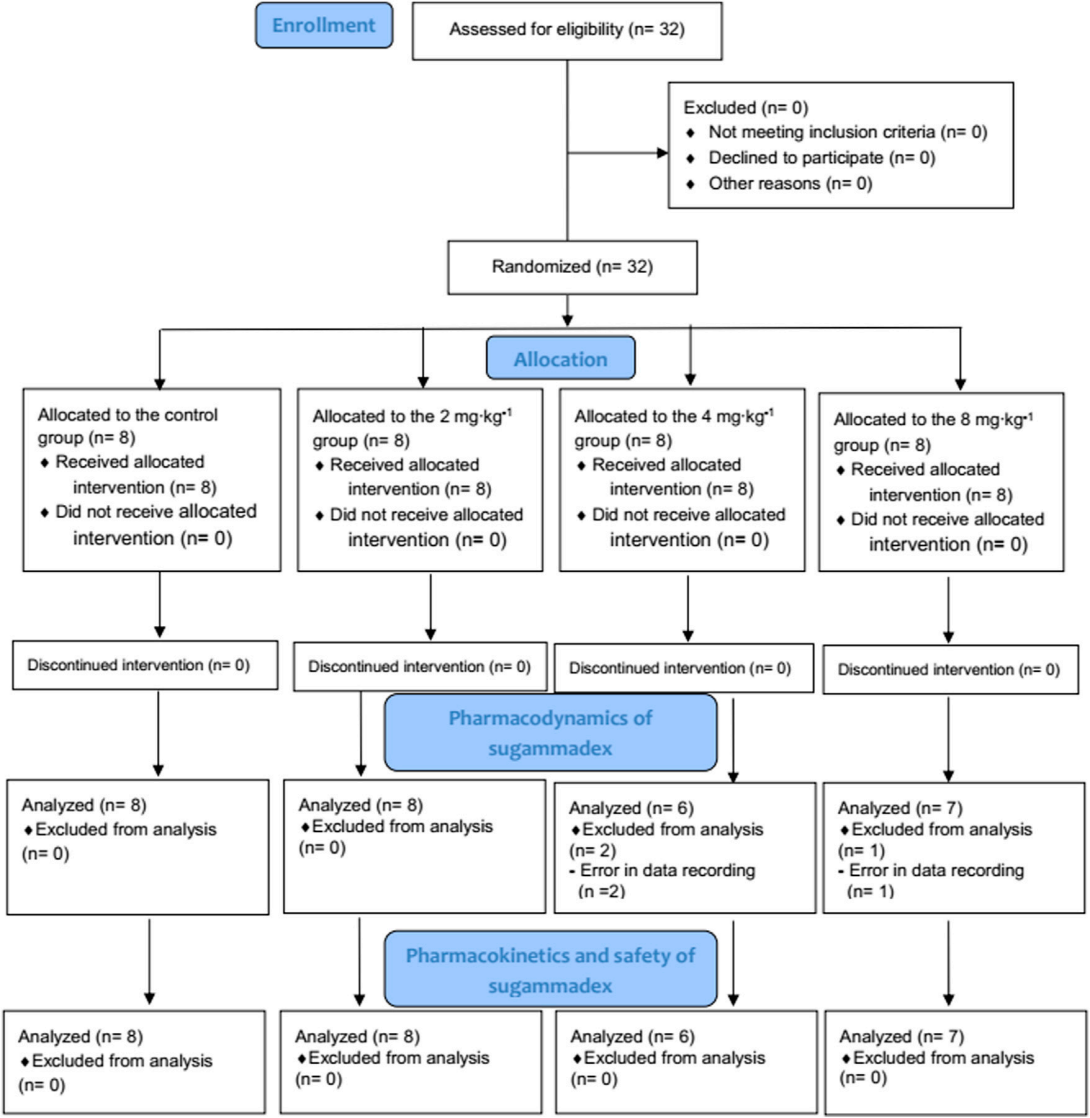
Consolidated Standards of Reporting Trials (CONSORT) flow diagram.

**FIGURE 2:**
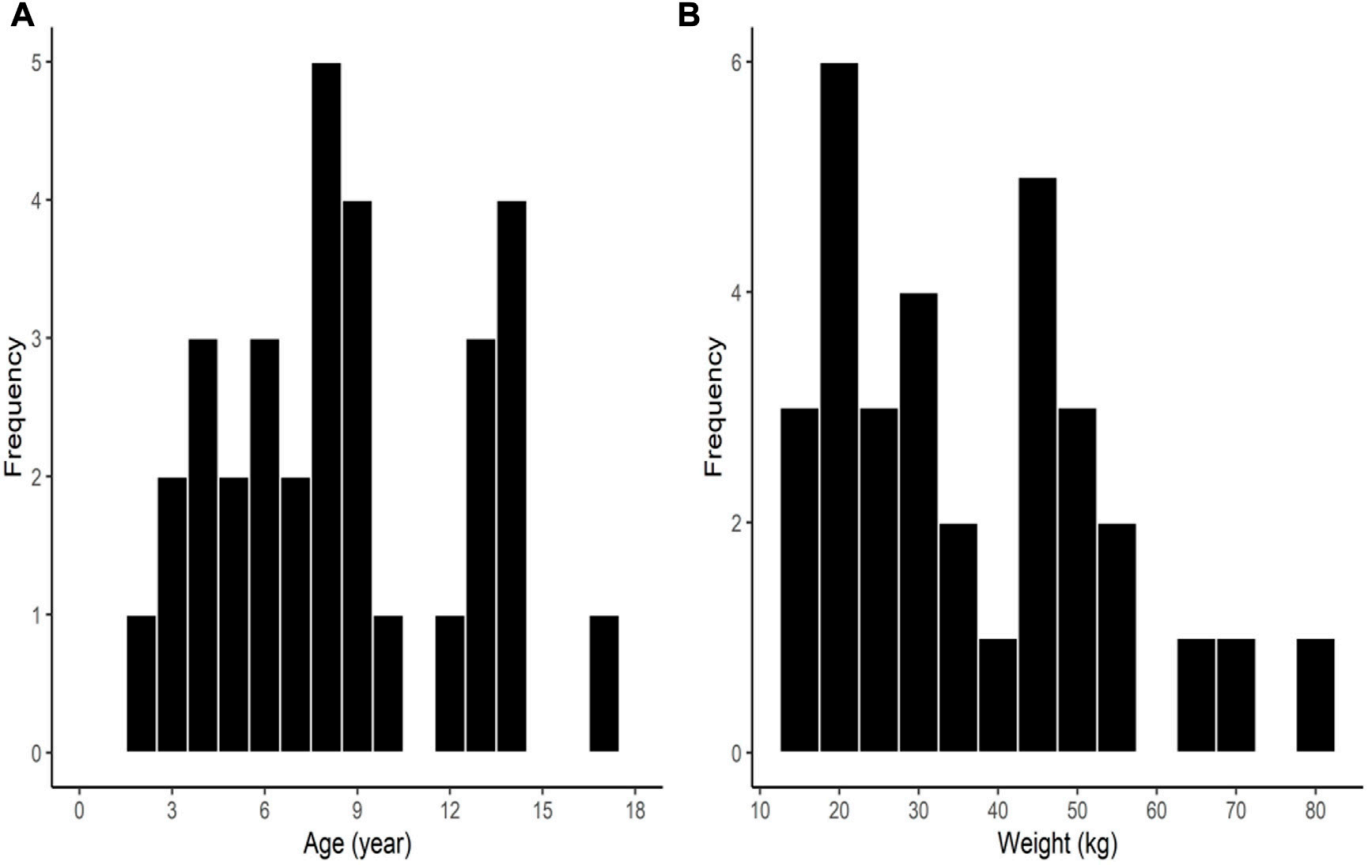
Histogram of the age **(A)** and body weight **(B)** of participants included in the analysis.

Among the 32 enrolled participants, we analyzed the pharmacodynamic data of 29 children. The remaining three participants were excluded owing to technical errors in data recording. For pharmacokinetic analysis, we obtained measurements of 257 plasma concentrations of rocuronium from 29 participants and 148 concentrations of sugammadex from 21 participants. Two concentration data points of rocuronium from each participant were removed from the analysis owing to the inevitable additional administration of rocuronium during the study. Figure 1 shows a flow diagram of the Consolidated Standards of Reporting Trials. We detected no significant differences among the groups with respect to baseline characteristics, which are shown in detail in Table 1. Distributions of the ages and body weights of the participants are shown as histograms in Figure 2.

**TABLE 1:** Demographic data.

	Control (neostigmine) (*n* = 8)	Sugammadex 2 mg/kg (*n* = 8)	Sugammadex 4 mg/kg (*n* = 6)	Sugammadex 8 mg/kg (*n* = 7)	*p*-value
Sex (male:female)	5:3	5:3	3:3	3:4	0.840
Age (years)	7.6 ± 3.1	10.5 ± 4.6	8.2 ± 4.5	8.4 ± 4.0	0.939
Height (cm)	128.8 ± 16.6	142.9 ± 27.2	130.0 ± 31.1	135.0 ± 22.7	0.857
Weight (kg)	31.0 ± 12.1	46.0 ± 21.1	30.3 ± 14.8	40.0 ± 18.6	0.659
Anesthesia time (min)	285.6 ± 54.9	339.4 ± 98.3	341.7 ± 63.9	310.0 ± 86.0	0.550
Operation time (min)	210 [167.5–235]	232.5 [195–280]	232.5 [205–290]	255 [207.5–282.5]	0.407
Type of surgery					0.814
Brain	6 (75%)	7 (87.5%)	4 (66.7%)	5 (71.4%)	
Spine	2 (25%)	1 (12.5%)	2 (33.3%)	2 (28.6%)	

Data are shown as median [interquartile range] values.

### 3.1 Efficacy of sugammadex

**FIGURE 3:**
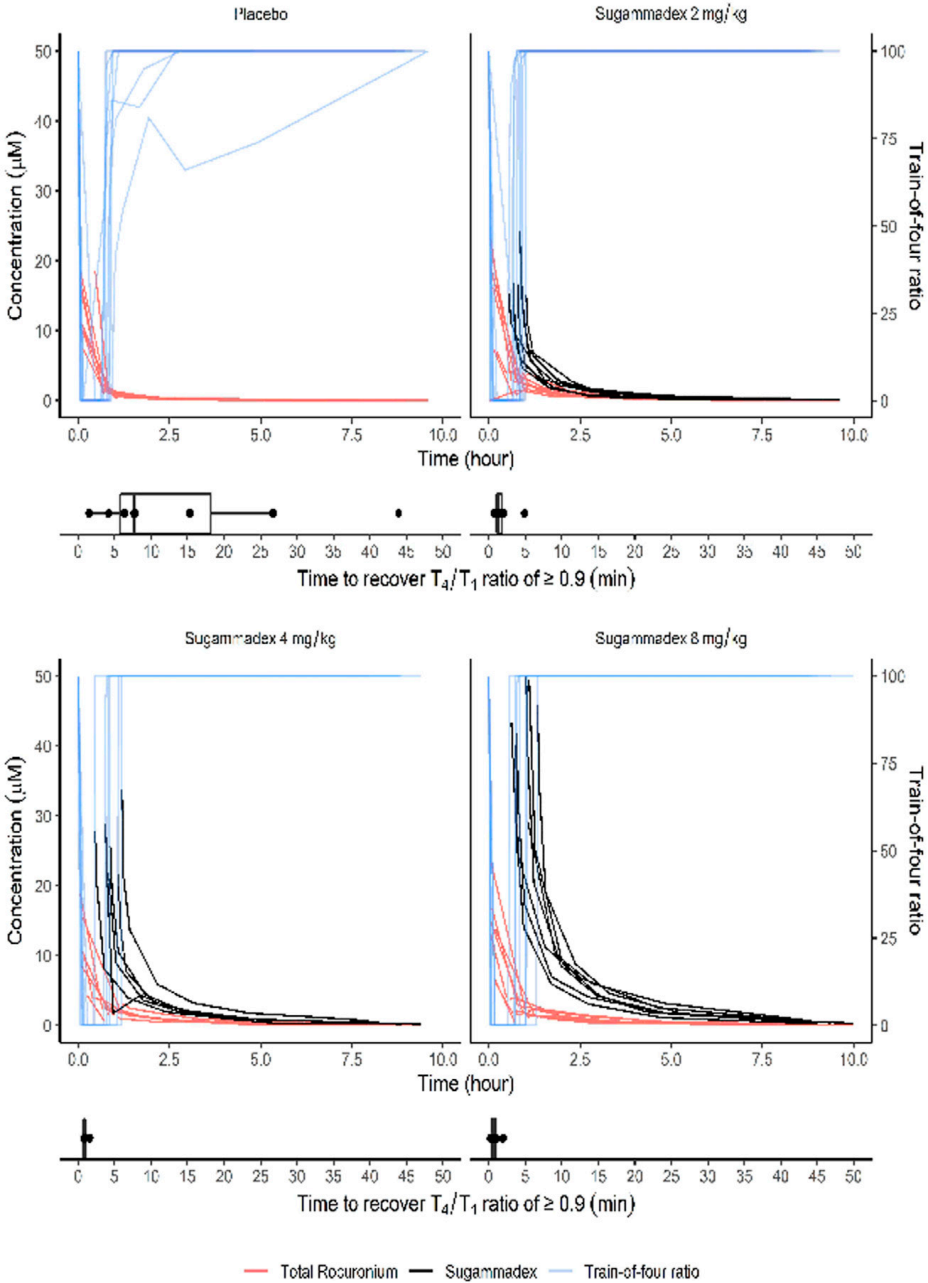
The T_4_/T_1_ ratio in response to train-of-four stimulation shown together with plasma molar concentrations of sugammadex and rocuronium. The box plots show the time from study drug administration to recovery of a T_4_/T_1_ ratio ≥0.9 in response to train-of-four stimulation. Molar concentrations were calculated based on the molecular weights of rocuronium (529.8 g/mol) and sugammadex (2,002 g/mol).

Average (±Standard deviation) time interval between rocuronium administration and sugammadex administration was 48.7 (±11.4) minutes. We found that the time [median (interquartile range)] from administration of sugammadex to achieving a T_4_/T_1_ ratio of 0.9 in response to train-of-four stimulation differed significantly among the groups (*p* < 0.001). For the control group, we recorded a recovery time of 7.7 (5.3–21.0) min, whereas that for participants in the 2, 4, and 8 mg/kg sugammadex groups was 1.3 (1.0–1.9), 0.9 (0.8–0.9), and 0.6 (0.4–1.0) min, respectively. In the *post hoc* analysis, we detected a significant difference between the control group and each of the groups treated with sugammadex (*p* = 0.002, 0.001, and 0.001 for the groups treated with 2, 4, and 8 mg/kg sugammadex, respectively). However, there were no significant differences among the groups treated with sugammadex. Figure 3 shows box plots of the recovery time for each group.

### 3.2 Pharmacokinetics

**FIGURE 4:**
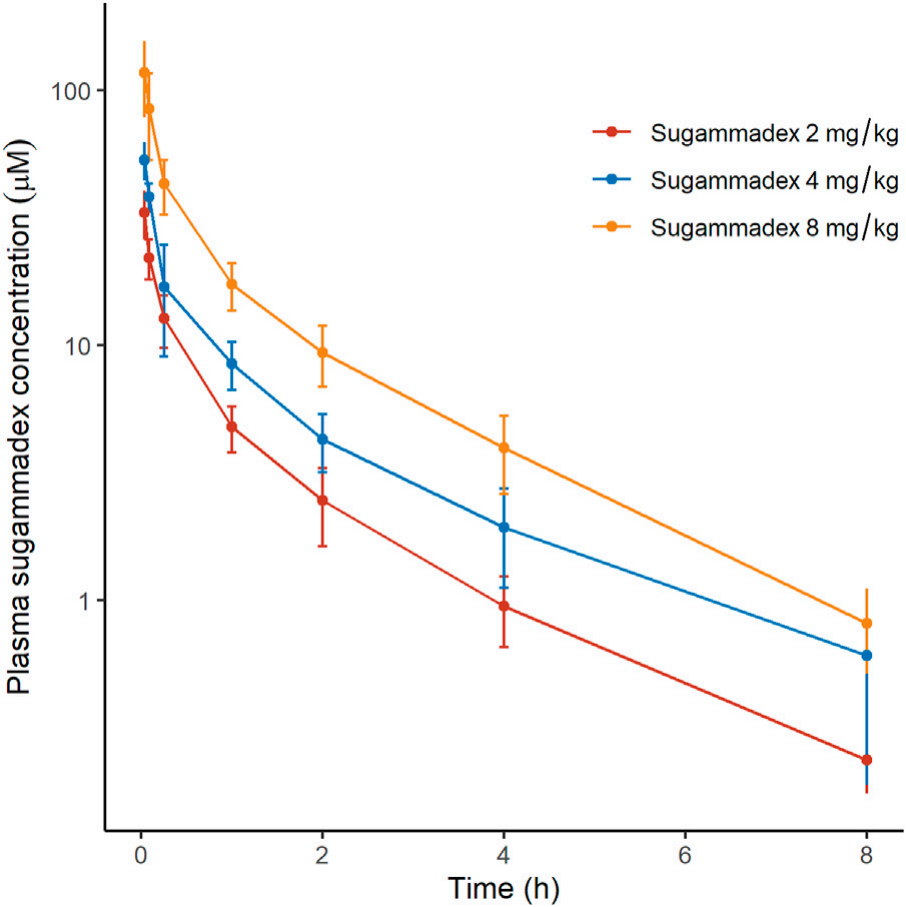
Plasma concentrations of sugammadex as a function of elapsed time after rocuronium administration, discriminated by allocated group. The *y*-axis is logarithmically scaled, and the error bars represent standard deviations.

Plasma sugammadex concentrations were found to be characterized by a biexponential decline, with a terminal half-life of 1.7–1.8 h. The systemic exposure to sugammadex was established to be proportional to the administered dose. Clearance (0.07 L/min) was consistent across dose levels and the volume of distribution ranged between 8.8 and 11.9 L (Figure 4; Table 2).

**TABLE 2:** Summary of pharmacokinetic parameters including comparison with previous study.

	Placebo			Sugammadex 2 mg/kg				Sugammadex 4 mg/kg				Sugammadex 8 mg/kg			
	Intense blockade^a^ (*n* = 10)	Moderate blockade^b^ (*n* = 8)	*p*-value	Intense blockade^a^ (*n* = 10)		Moderate blockade^b^ (*n* = 8)	*p*-value	Intense blockade^a^ (*n* = 10)		Moderate blockade^b^ (*n* = 6)	*p*-value	Intense blockade^a^ (*n* = 10)	Moderate blockade^b^ (*n* = 7)	*p*-value
Sugammadex														
C_max_ (μg/mL)		—		28.4 ± 9.6		33.2 ± 7.2	0.315	58.2 ± 12.5		53.3 ± 9.1	0.793	118.9 ± 13	117.3 ± 38.8	0.109
AUC_last_ (h·μg/mL)		—		13.6 ± 3.7		19.4 ± 4.5	0.633	28 ± 6.3		31.5 ± 8.1	0.118	51 ± 9.6	71.5 ± 18.2	0.962
AUC_inf_ (h·μg/mL)		—		14.2 ± 3.8		20.2 ± 4.7	0.633	29.1 ± 7		33.2 ± 9.0	0.147	53.9 ± 13	73.5 ± 18.8	0.813
T_max_ (h)		—		0.03 [0.02–0.03]		0.02 [0.02–0.03]	0.227	0.02 [0.02–0.03]		0.03 [0.03–0.04]	0.115	0.03 [0.02–0.03]	0.03 [0.02–0.03]	0.353
t_1/2_ (h)		—		1.5 ± 0.4		1.8 ± 0.5	0.146	1.7 ± 0.2		1.7 ± 0.3	0.635	1.6 ± 0.2	1.7 ± 0.1	0.088
V_z_ (L)		—		9.9 ± 6.8		11.9 ± 6.1	0.173	10.6 ± 2.8		8.8 ± 4.1	0.562	9.7 ± 5.1	11.0 ± 3.3	0.364
CL (L/min)		—		0.07 ± 0.04		0.07 ± 0.03	0.633	0.08 ± 0.03		0.06 ± 0.02	0.147	0.07 ± 0.03	0.07 ± 0.02	0.813
Rocuronium														
C_max_ (μg∙/mL)	6.8 ± 3.1	7.2 ± 2.3	0.237	5.3 ± 1.9		7.5 ± 4.1	0.633	8.0 ± 2.1		6.0 ± 2.8	0.635	5.4 ± 3.0	7.1 ± 3.1	0.813
AUC_last_ (h·μg/mL)	2.9 ± 0.9	3.1 ± 0.8	0.146	3.7 ± 1.0		4.7 ± 1.6	0.237	4.8 ± 1.1		4.5 ± 1.7	0.958	4.0 ± 1.4	5.1 ± 1.7	0.601
AUC_inf_ (h·μg/mL)	2.9 ± 0.9	3.2 ± 0.8	0.146	3.7 ± 1.0		4.7 ± 1.6	0.274	4.9 ± 1.1		4.6 ± 1.7	0.875	4.3 ± 1.8	5.2 ± 1.7	0.364
T_max_ (h)	0.08 [0.03–0.22]	0.09 [0.05–0.47]	0.446	0.1 [0.07–0.25]		0.11 [0.05–0.98]	1.000	0.07 [0.03–0.13]		0.13 [0.05–0.25]	0.018	0.12 [0.03–0.43]	0.08 [0.05–0.17]	0.883
t_1/2_ (h)	1.1 ± 0.4	1.5 ± 0.9	0.315	1.2 ± 0.3		1.6 ± 0.3	0.006	1.2 ± 0.2		1.7 ± 0.6	0.016	1.3 ± 0.2	1.7 ± 0.1	0.000
V_z_ (L)	21.4 ± 10.8	19.9 ± 12.1	0.762	14.5 ± 11.8		23.2 ± 11.9	0.034	10.8 ± 3.5		15.9 ± 6.1	0.181	13.0 ± 5.9	19.7 ± 6.5	0.033
CL (L/min)	0.22 ± 0.07	0.17 ± 0.07	0.146	0.13 ± 0.08		0.17 ± 0.07	0.274	0.11 ± 0.04		0.11 ± 0.05	0.875	0.12 ± 0.05	0.13 ± 0.04	0.364

^a^ Data from our previous study (Clin Transl Sci. 2023 January; 16 (1):92-103. Doi: 10.1111/cts.13429.).

^b^ Data from current study.

^†^Kruskal–Wallis test. Note: Data are presented as the mean ± standard deviation, except for T_max_, for which median [minimum−maximum] values are presented. Abbreviations: C_max_, maximum plasma concentration; AUC, area under the plasma concentration-time curve; AUC_last_, AUC, from time zero to the last observable concentration; AUC_inf_, AUC, from time zero to infinity; T_max_, time to reach the maximum plasma concentration; t_1/2_, terminal-phase elimination half-life; V_z_, terminal-phase volume of distribution; CL, clearance.

Age group was found to be significantly associated with pharmacokinetics parameters, with reductions in dose-normalized C_max_, AUC_last_, and AUC_inf_ being observed with increasing age. In contrast, both the clearance and volume of distribution were observed to be higher in older children, whereas the elimination half-life was consistent among all age groups. Subgroup analysis of data from our previous study (Ji et al., 2023) based on age group showed similar results (Table 3).

**TABLE 3:** Summary of pharmacokinetic parameters of sugammadex according to age.

	Age group	Age group	Age group	*p*-value^†^
	2 to <6 (yrs)	6 to <12 (yrs)	12 to <17 (yrs)	
Reversal for intense blockade^a^	(*n* = 8)	(*n* = 14)	(*n* = 8)	
Dose-normalized C_max_ (μg/mL/mg)	0.8 ± 0.1	0.6 ± 0.3	0.3 ± 0.1	0.0005
Dose-normalized AUC_last_ (h·μg/mL/mg)	0.4 ± 0.1	0.3 ± 0.1	0.2 ± 0.0	0.0009
Dose-normalized AUC_inf_ (h·μg/mL/mg)	0.4 ± 0.1	0.3 ± 0.1	0.2 ± 0.1	0.0008
t_1/2_ (h)	1.7 ± 0.1	1.4 ± 0.3	1.8 ± 0.3	0.0309
V (L)	7.0 ± 1.8	8.3 ± 3.1	16.2 ± 4.8	0.0005
CL (L/min)	0.05 ± 0.01	0.07 ± 0.02	0.11 ± 0.03	0.0008
Reversal for moderate blockade^b^	(*n* = 5)	(*n* = 8)	(*n* = 8)	
Dose-normalized C_max_ (μg/mL/mg)	0.7 ± 0.3	0.5 ± 0.1	0.3 ± 0.1	^b^0.0031
Dose-normalized AUC_last_ (h·μg/mL/mg)	0.4 ± 0.1	0.3 ± 0.1	0.2 ± 0.0	^b^0.0027
Dose-normalized AUC_inf_ (h·μg/mL/mg)	0.4 ± 0.1	0.3 ± 0.1	0.2 ± 0.0	^b^0.0027
t_1/2_ (h)	1.6 ± 0.3	1.8 ± 0.1	1.8 ± 0.5	0.7670
V (L)	6.4 ± 3.5	9.8 ± 2.6	14.4 ± 4.5	^b^0.0095
CL (L/min)	0.04 ± 0.02	0.06 ± 0.02	0.09 ± 0.02	^b^0.0027

^a^ Data from our previous study (Clin Transl Sci. 2023 January; 16 (1):92-103. Doi: 10.1111/cts.13429.).

^b^ Data from current study.

^†^Kruskal–Wallis test. Abbreviations: C_max_, maximum plasma concentration; AUC, area under the plasma concentration-time curve; AUC_last_, AUC, to the last measurable concentration; AUC_inf_, AUC, extrapolated to infinity; t_1/2_, terminal-phase half-life; V, volume of distribution; CL, clearance.

**FIGURE 5:**
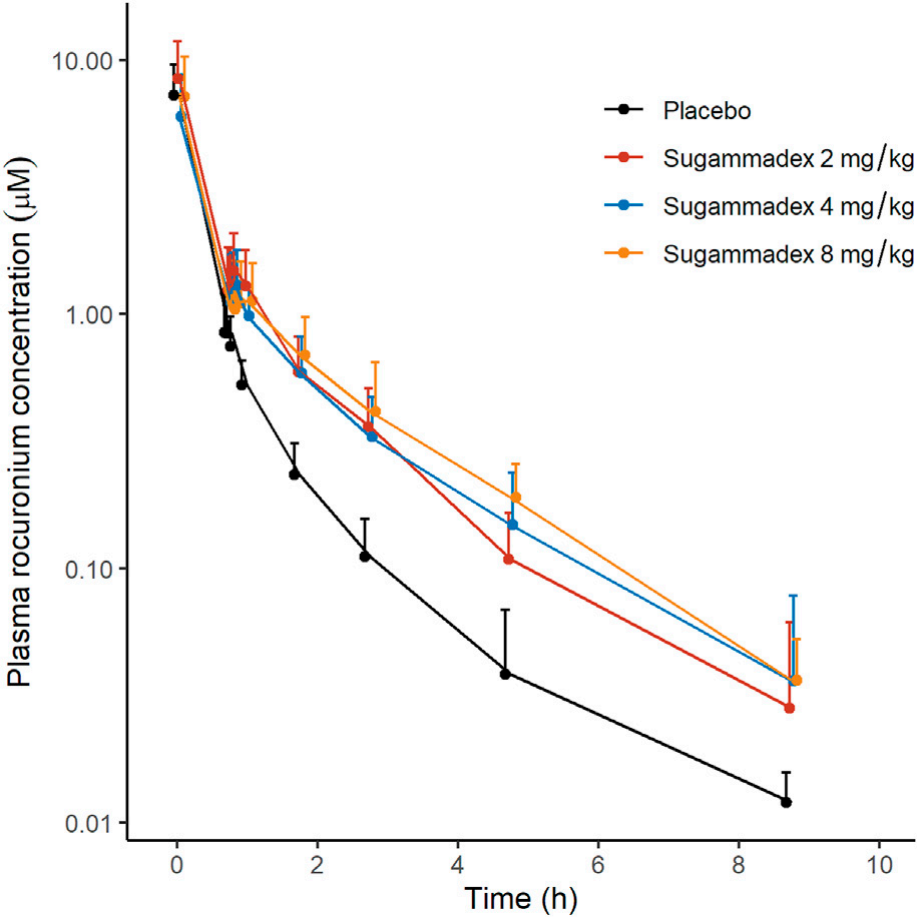
Plasma concentrations of rocuronium as a function of elapsed time after rocuronium administration, discriminated by allocated group. The *y*-axis is logarithmically scaled, and the error bars represent standard deviations.

Plasma rocuronium concentration was also characterized by a biexponential decline, with a half-life of 1.6–1.7 h. Systemic exposure to rocuronium was comparable between the control and sugammadex groups, with clearance and volume values ranging between 0.11 and 0.16 L/min and 15.9 and 21.1 L, respectively (Figure 5; Table 2). Figure 3 shows the molar concentrations of sugammadex and rocuronium, along with the T_4_/T_1_ ratio of the individual participants.

There were no statistically significant difference between this study and our previous study in any of the parameters for sugammadex. For rocuronium, C_max_ and AUCs were similar, while *t*_1/2_ and V_z_ tended to show larger values in this study (Table 2).

### 3.3 Safety profiles of sugammadex

During the period between administration of the study drug and 24 h after the end of surgery, 20 of the 29 participants experienced a range of adverse side effects, including fever, hypothermia, hypotension, hypertension, tachycardia, bradycardia, headache, nausea, and vomiting. Among these, two hypotensive events were considered to be associated with the use of sugammadex, although these were self-limiting and no subsequent intervention was necessary. Other adverse events were considered to be surgery-related rather than attributable to the use of a neuromuscular reversal agent. Detailed profiles of the adverse events are presented in Table 4.

**TABLE 4:** Summary of adverse events after sugammadex administration.

Group	Control (*n* = 8)	Sugammadex	Sugammadex	Sugammadex
		2 mg/kg (*n* = 8)	4 mg/kg (*n* = 6)	8 mg/kg (*n* = 7)
Number of participants with any adverse event	6 (75%)	6 (75%)	5 (83.3%)	3 (42.9%)
Fever	2 (25%)	—	—	1 (14.3%)
Hypothermia	1 (12.5%)	—	—	—
Hypotension	1 (12.5%)	2 (25%)	3 (50%)	1 (14.3%)
Hypertension	1 (12.5%)	1 (12.5%)	2 (33.3%)	—
Tachycardia	1 (12.5%)	2 (25%)	1 (16.7%)	—
Bradycardia	—	1 (12.5%)	—	—
Headache	—	—	1 (16.7%)	—
Nausea	—	1 (12.5%)	—	—
Vomiting	—	—	1 (16.7%)	1 (14.3%)
Number of participants who received any intervention for adverse events	—	—	—	—
Number of adverse events relevant to study drug	—	1 (12.5%)	1 (16.7%)	—
Hypotension		1 (12.5%)	1 (16.7%)	

Adverse events were recorded from the administration of sugammadex to 24 h after the end of surgery.

## 4 Discussion

In this study, we evaluated the efficacy of sugammadex in reversing rocuronium-induced neuromuscular blockade in Korean children, in the context of a conventional reversal at reappearance of T_2_ in response to train-of-four stimulation. In addition, we performed a non-compartmental analysis of sugammadex and rocuronium according to each sugammadex dose group. One type of adverse event was suspected to be related to sugammadex use.

Previous studies have investigated the pharmacodynamics of sugammadex in the context of the reversal of moderate neuromuscular blockade in children. The median recovery time of 1.3 min from 2 mg/kg sugammadex observed in the present study is comparable to that previously reported (Plaud et al., 2009; Kleijn et al., 2011; Voss et al., 2022). In response to higher doses, we established that the reversal times were similar to or less than those recorded following the administration of 2 mg/kg sugammadex. As the median recovery time of 1.3 min is considered sufficiently short, we can conclude that the recovery profile of sugammadex in Korean children is similar to that reported in the literature. In this regard, Herring et al. (Herring et al., 2017) reported a pooled analysis of adult data, which revealed a geometric mean of recovery time of 1.9 min in response to the administration of 2 mg/kg sugammadex, compared with a value of 10.6 min recorded in those treated with neostigmine. The median recovery time of 1.3 min recorded in the present study is accordingly comparable to that obtained by these authors, thereby indicating that a dose of 2 mg/kg sugammadex would be suitable for use in reversing moderate neuromuscular blockade in children.

In contrast to pharmacodynamics, pharmacokinetic analyses of sugammadex in children have rarely been reported. Among those studies that have been conducted is a recent phase IV trial, in which the pharmacokinetics of 2 and 4 mg/kg sugammadex were evaluated in 40 pediatric patients (Voss et al., 2022), the findings of which revealed that pediatric patients aged between 6 and 17 years were characterized by similar systemic exposure to their adult counterparts (Voss et al., 2022). Contrastingly, pediatric patients between the ages 2 and 6 years were observed to have between 25% and 40% lower systemic exposure (Voss et al., 2022). Overall, the pharmacokinetic profiles of these patients were similar to those characterized in our Korean pediatric study.

We established that the age-based pharmacokinetic parameters of sugammadex were closely correlated with pediatric ontogeny, with the values of both the volume of distribution and clearance being high in older children. Indeed, the clearance of sugammadex in adolescent patients (i.e., those aged between 12 and 17) was similar to that reported for adult patients (Keating, 2016). Given that sugammadex is primarily eliminated via renal excretion, the observed changes in clearance are assumed to reflect the increasing size and maturation of kidney function with increasing age (Anderson, 2012). The volume of distribution was found to increase to a similar extent, as indicated by an unchanged elimination half-life. However, in this regard, the ontogeny of hepatic function also deserves consideration, given that sugammadex recovery time has been demonstrated to be slower after liver transplantation, even though the metabolism of sugammadex is minimal (Deana et al., 2020). This result is also consistent with our previous study on reversal of deep neuromuscular blockade. Considering these, there is possibility that less dose may be needed for younger children to obtain similar effect. Further pharmacodynamics studies for effect of age on pharmacodynamics of sugammadex may help.

We found that patients in the groups receiving sugammadex were characterized by slightly higher plasma concentrations of rocuronium than those recorded in control group (Figure 5). Given that the complex of rocuronium and sugammadex is indistinguishable from free rocuronium, it is assumed that plasma concentrations will not undergo a marked decline, even after the administration of sugammadex. In addition, Ploeger et al. have reported that the observed plasma concentrations of rocuronium can increase following the administration of sugammadex (Ploeger et al., 2009), which they attribute to the movement of free rocuronium from the tissue compartment to the plasma compartment to establish a steady state, as free rocuronium binds to sugammadex. In a study on the effects of sugammadex on the pharmacokinetics of rocuronium, Gijsenbergh et al. reported a dose-dependent increase in the AUC_inf_ of rocuronium with increasing sugammadex concentration (Gijsenbergh et al., 2005). As shown in Figure 5, we detected notable differences among groups in this regard. Furthermore, in our non-compartmental analysis, we detected increases in AUC_last_ and AUC_inf_ of rocuronium in response to the administration of sugammadex, although the differences were not statistically significant (*p* = 0.765, 0.089, 0.064 for C_max_, AUC_last_, AUC_inf_, respectively). We speculate that this inconsistency could be attributable to the small number of participants assessed in this study. These changes were also observed in our previous study on reversal of intense blockade, and the differences were significant (Ji et al., 2023). The C_max_ of rocuronium should not differ since the highest concentration of rocuronium is reached prior to the administration of sugammadex.

Differences were observed on *t*_1/2_ and V_z_ of rocuronium between this study and our previous study. This may be due to difference of the timing of sugammadex administration in both studies (48.7 min in this study vs. 14.9 min in previous study, *p* < 0.001).

As potential adverse effects of sugammadex, bradycardia, QT interval prolongation, atrioventricular block, hypotension, atrial fibrillation, hypersensitivity, and anaphylaxis have been reported (de Kam et al., 2018; Min et al., 2018; Kapoor, 2020). Among these, bradycardia has been reported to occur in approximately 8% of children administered sugammadex, although the incidence is lower than that observed following the administration of neostigmine (Alsuhebani et al., 2020). In the present study, one (4.8%) of the 21 participants who received sugammadex developed bradycardia, although in this case, the event was considered to be unrelated to the use of sugammadex, as it occurred approximately 9 h after administration. Moreover, we observed two occurrences of hypotension following the administration of sugammadex, although we were unable to establish whether these were associated with the administration of sugammadex or anesthetic drugs. Nevertheless, given that no intervention was necessary for either of these events, they were not considered to be serious.

Our study had some limitations. Firstly, we did not establish a pharmacokinetic–pharmacodynamic model of sugammadex for the reversal of neuromuscular blockade, as has been done in some previous studies (Ploeger et al., 2009; Kleijn et al., 2011). For the purpose of this study, we decided not to include a pharmacokinetic model, given that precise pharmacokinetic modeling is difficult, owing to the inability to discriminate the rocuronium–sugammadex complex from the free forms of these drugs. In addition, as recovery generally occurred within 2 min, it was difficult for multiple blood samplings during this interval to construct a viable pharmacokinetic–pharmacodynamic model. Secondly, given that we enrolled a relatively small number of participants, we did not include a subgroup analysis of pharmacodynamics according to the age spectrum. Accordingly, further efficacy studies involving a larger number of participants are required.

In conclusion, we report a pharmacokinetic and efficacy study of sugammadex administered for the reversal of moderate neuromuscular blockade in Korean children, along with an analysis based on age group. We accordingly established that a 2 mg/kg dose of sugammadex appears to be safe and effective in reversing rocuronium-induced neuromuscular blockade on reappearance of the T_2_ twitch in response to train-of-four stimulation in Korean children older than 2 years of age. Also, pharmacokinetic parameters of sugammadex differed according to age. Further studies, including post-marketing surveillance and study on effect of age, are needed to enable a more robust conclusion regarding the efficacy and safety of this drug.

## Data Availability

The raw data supporting the conclusion of this article will be made available by the authors, without undue reservation.
